# FOXO3 LONGEVITY GENOTYPE MITIGATES THE IMPACT OF HYPERTENSION ON RISK OF INTRACEREBRAL HEMORRHAGE

**DOI:** 10.1093/geroni/igac059.958

**Published:** 2022-12-20

**Authors:** Kazuma Nakagawa, Randi Chen, Steven Greenberg, Webster Ross, Bradley Willcox, Timothy Donlon, Brian Morris, Kamal Masaki

**Affiliations:** Kuakini Medical Center, Honolulu, Hawaii, United States; Kuakini Medical Center, Honolulu, Hawaii, United States; Massachusetts General Hospital, Boston, Massachusetts, United States; Pacific Health Research and Education Institute, Honolulu, Hawaii, United States; Kuakini Medical Center, Honolulu, Hawaii, United States; Kuakini Medical Center, Honolulu, Hawaii, United States; Kuakini Medical Center, Honolulu, Hawaii, United States; Kuakini Medical Center, Honolulu, Hawaii, United States

## Abstract

The longevity-associated FOXO3 G allele of SNP rs2802292 is associated with protection against mortality, however the impact of FOXO3 genotype on the association between hypertension and risk of intracerebral hemorrhage (ICH) has not been assessed. Therefore, we utilized the Kuakini Honolulu Heart Program (KHHP), a prospective population-based cohort of Japanese American men living in Hawaii that began in 1965 to study this relation. After excluding baseline prevalent stroke and those missing FOXO3 data, 6,469 men were included in the analysis. Age-adjusted prevalence of ICH by hypertension was assessed for the whole cohort after stratifying by FOXO3 genotype. Cox regression models, adjusted for age, cardiovascular risk factors, FOXO3 and APOE genotypes, were utilized to assess relative risk of hypertension on ICH. All models were created for the whole cohort and stratified by FOXO3 genotype, namely G allele carriers versus TT genotype. Overall, 183 subjects developed ICH over the 34-year follow-up period. Age-adjusted ICH prevalence was 0.90 versus 1.32 per 1,000 person-years follow-up in those without and with hypertension, respectively (p=0.002). After stratifying by FOXO3 genotype, this association was no longer significant in G allele carriers. In the whole cohort, hypertension was an independent predictor of ICH (RR=1.70, 95%CI 1.25, 2.32; p=0.0007). In stratified analyses, hypertension remained an independent predictor of ICH among the FOXO3 TT allele group (RR=2.02, 95% CI 1.33, 3.07; p=0.001), but not in FOXO3 G allele carriers (RR=1.39, 95% CI 0.88, 2.19; p=0.15). Thus, the longevity/resilience-associated FOXO3 G allele may mitigate the impact of hypertension on ICH risk in this population. Further study is needed in other populations.

